# Distilling AI Workforce-Readiness Competencies for U.S. Pharmacists from Job Advertisements: Implications for Pharmacy Education

**DOI:** 10.3390/pharmacy14060138

**Published:** 2026-09-20

**Authors:** Ashim Malhotra, Alvin Cheung

**Affiliations:** 1 Department of Pharmaceutical and Biomedical Sciences, College of Pharmacy, California Northstate University, 9700 West Taron Drive, Elk Grove, CA 95758, USA; 2 California Northstate University, 9700 West Taron Drive, Elk Grove, CA 95758, USA

**Keywords:** artificial intelligence, pharmacist workforce, competencies, pharmacy education, employment advertisements, clinical reasoning, medication safety, clinical pharmacology

## Abstract

Artificial intelligence (AI) is entering medication-use and drug-development workflows, but the AI-specific capabilities needed for pharmacist workforce readiness remain poorly characterized. This pilot environmental scan and qualitative content analysis examined publicly accessible United States employment advertisements covering 1 January 2023 through 10 August 2026. Thirty-four distinct candidate requisitions were audited; 31 met operational criteria. Ten explicitly required AI-related work and formed the primary analytic sample, while 21 AI-enabling informatics, electronic-health-record, automation, analytics, and clinical-decision-support roles were retained as contextual comparators. AI-explicit employers sought pharmacists or PharmD-eligible professionals to evaluate model-generated clinical content, correct unsafe reasoning, construct prompts and cases, curate datasets and reference answers, verify claims, apply structured evaluation frameworks, validate dosing and pharmacokinetic reasoning, provide iterative model feedback, and implement AI-enabled clinical-pharmacology use cases. These activities were synthesized into five AI workforce-readiness competencies: AI-output evaluation and validation; AI systems and workflow literacy; prompt and evaluation-task design; AI performance assessment and improvement; and responsible AI implementation and governance. The framework distinguishes these AI-specific capabilities from the pharmacotherapy, medication-safety, evidence-appraisal, and quantitative expertise required to exercise them safely. These preliminary workforce signals support staged curricular development while requiring larger prospective and stakeholder-validated studies.

## 1. Introduction

Artificial intelligence (AI) is moving from a speculative technology to an operational component of healthcare. Machine-learning systems are used for prediction, image interpretation, workflow prioritization, and clinical decision support, while generative AI systems can produce medication information, patient education, clinical documentation, and apparently, reasoned answers to therapeutic questions. Pharmacy is especially exposed to both the advantages and risks of this transition because medication decisions depend on accurate integration of patient characteristics, drug properties, calculations, evidence, monitoring, and workflow context. The International Pharmaceutical Federation (FIP) has consequently called for a digitally literate pharmacy workforce capable of integrating AI without surrendering critical thinking or professional judgment [1]. The American Society of Health-System Pharmacists (ASHP) similarly assigns pharmacists active roles in the design, implementation, validation, governance, and surveillance of AI used across medication-use systems [2].

The competency problem is more specific than whether pharmacists know how to use a chatbot. AI systems may produce fluent but incorrect recommendations, overlook contraindications, mishandle units, cite nonexistent evidence, or fail to recognize when essential patient data are missing. Bias may enter through unrepresentative training data, clinical evidence, labels, proxy variables, or workflow design. FIP therefore states that AI processes related to healthcare require pharmacist supervision and validation [3]. Broader health-professions research has proposed competencies in foundational AI knowledge, social and ethical implications, AI-enhanced clinical encounters, evidence-based evaluation, workflow analysis, and practice-based improvement [4]. Yet, a scoping review found remarkably little research measuring the competencies clinicians need to use AI effectively [5]. Pharmacy-specific empirical evidence about what employers are presently asking pharmacists to do remains even thinner.

Existing pharmacy literature largely approaches AI from three directions. First, professional statements and practice guidance describe principles for responsible adoption and emphasize patient safety, oversight, transparency, data quality, and accountability [1,2,3,6,7]. Second, practice literature maps possible applications in clinical and operational pharmacy, such as decision support, pharmacovigilance, inventory management, adherence, documentation, and automation [8], while emerging risk analyses examine potential harms to patients from inaccurate, biased, or poorly governed AI use [9]. Third, educational studies examine pharmacy students’ attitudes or introduce AI-supported learning activities [10,11,12]. These bodies of work are important but do not directly answer a workforce-readiness question: when employers explicitly recruit pharmacists for AI-related work, what work are they asking them to perform?

This gap matters because competency frameworks can become aspirational inventories disconnected from actual occupational change. Conversely, waiting until AI-related duties are common in conventional advertisements may leave colleges and schools of pharmacy reacting after workflows have already shifted. A workforce scan can function as an early warning mechanism: it captures weak but concrete signals, distinguishes newly purchased expertise from broad technological enthusiasm, and reveals the form in which clinical knowledge is becoming valuable to employers. For an emerging field, the meaning of a small number of highly specific roles may therefore be more informative than an uncritical count of every advertisement mentioning technology.

Employment advertisements provide a useful, although incomplete, window into emerging demand. Unlike aspirational competency frameworks, an advertisement represents an organization’s attempt to acquire capabilities for a current role. Job-advertisement analysis has been used to identify changing competency requirements in rapidly developing fields because it links occupational titles, qualifications, tasks, and employer language at a particular point in time [13]. It does not establish what every practitioner should know, and online postings are not a census of employment. Nevertheless, carefully audited advertisements can reveal which capabilities organizations are beginning to purchase before those capabilities become standardized in professional frameworks or curricula.

For pharmacy, an additional distinction is essential. Many pharmacist positions require informatics, electronic health records (EHRs), automation, data analytics, system validation, or clinical decision support (CDS) capabilities. These capabilities may enable safe AI implementation, but they are not themselves proof that the employer requires AI competency. Combining AI-explicit and informatics advertisements would inflate the apparent prevalence of AI demand and blur two analytically different workforce signals. The first concerns direct work with AI models or AI-supported practice; the second concerns the digital and sociotechnical infrastructure within which future AI may operate.

This distinction also protects the educational interpretation. Informatics capability is likely a prerequisite for many AI-enabled workflows, but it cannot substitute for the ability to interrogate model reasoning, recognize hallucination or bias, validate patient-specific calculations, and decide when an output should be rejected. Conversely, AI literacy without understanding EHR data, medication workflows, interfaces, and automation may remain detached from actual practice. A credible competency model must therefore identify where the two domains overlap while preserving the empirical boundary between them.

This pilot study therefore examined publicly accessible U.S. pharmacist employment advertisements using a two-tier design. The primary objective was to identify and interpret competencies requested in advertisements that explicitly described AI-related work. A secondary objective was to characterize AI-enabling informatics advertisements as a contextual comparator without treating them as direct evidence of AI demand. Finally, the study translated observed tasks into evidence-anchored, future-facing competencies for pharmacy education. The guiding question was: What do currently retrievable employment advertisements reveal about the emerging role of pharmacists in evaluating, training, implementing, and governing AI-supported medication reasoning?

## 2. Materials and Methods

### 2.1. Study Design

This study used a retrospective environmental scan and inductive qualitative content analysis of publicly accessible employment advertisements. The unit of analysis was a distinct employment requisition rather than each webpage, search result, or duplicate location-specific posting. Advertisements explicitly describing AI, generative AI, machine learning, model training, model evaluation, or comparable model-focused work formed the primary analytic set. A separate AI-enabling comparator set comprised pharmacist roles involving informatics, electronic health records (EHRs), clinical decision support (CDS), automation, analytics, system validation, or digital governance without an explicit AI duty. Comparator advertisements contextualized the implementation infrastructure surrounding AI but were not counted as direct evidence of AI competency demand. The qualitative content-analysis design was informed by established methodological guidance [14]. Reporting of the qualitative components was structured using the Standards for Reporting Qualitative Research (SRQR) [15].

### 2.2. Coverage Period and Search Dates

Advertisements were eligible if their displayed posting or availability fell between 1 January 2023 and 10 August 2026. The start date was selected as the first full calendar year after the public release of ChatGPT on 30 November 2022, when generative AI became broadly accessible and could plausibly begin influencing occupational language. Searches, verification, and the final expansion audit were conducted from 7 through 10 August 2026. Because expired advertisements are routinely deleted or de-indexed, this interval defines the intended coverage window rather than complete historical capture. The dataset was treated as a retrievable open-web sample, not a census or a time-trend series.

### 2.3. Information Sources and Source-Selection Rationale

Sources represented major open-access pathways for U.S. pharmacist employment—ASHP CareerPharm, the APhA Career Center, the ACCP Career Center, AMCP resources, AACP and HigherEdJobs resources, USAJOBS, Indeed, LinkedIn Jobs, Health eCareers, direct employer applicant-tracking systems and career pages—and open AI-training platforms. Direct sources included government, university, health-system, Workday, Oracle Recruiting, pharmaceutical-industry, recruiter, and platform pages. General job services and professional boards supported discovery and corroboration; original employer or government pages were retained as canonical sources whenever available. This open-web design permits replication without a paid labor-market database but may underrepresent advertisements accessible only through subscriptions, login barriers, or historical archives.

### 2.4. Search Concepts and Query Construction

The prespecified search inventory contained 336 generated search strings. This total resulted from systematically combining six source groups with four pharmacist identifiers—‘pharmacist,’ ‘PharmD,’ ‘doctor of pharmacy,’ and ‘RPh’—and 14 AI-explicit or AI-enabling technology terms (6 source groups × 4 pharmacist identifiers × 14 technology terms = 336 generated term-and-source combinations). The inventory served as a query-generation and search-planning procedure; it does not establish that all 336 strings were executed, and it does not represent 336 advertisements or 336 conceptually distinct research questions. Actual candidate discovery used targeted, source-specific searches, supplemented by high-recall site-restricted searches using job titles, employer names, requisition numbers, and phrases such as ‘pharmacist AI trainer,’ ‘clinical pharmacist AI evaluator,’ ‘PharmD generative AI,’ ‘pharmacist machine learning,’ and ‘clinical pharmacology artificial intelligence.’ Search dates, candidate URLs, verification decisions, and corrections were retained in versioned files. Accordingly, the study is characterized as a targeted pilot environmental scan rather than an exhaustive or systematic search.

### 2.5. Prespecified Query-Generation Procedure

The following executable Python code (Box 1) generated the inventory of 336 term-and-source combinations. The code generates potential search strings; it does not scrape websites, document query execution, or establish that every generated string was executed.

Box 1 **Executable Python Code.**

"""Generate the prespecified search-query inventory for the pharmacist AI job scan.

 

This script creates search strings; it does not scrape websites and does not

claim that every generated string was executed. Output is tab-delimited.

"""

from itertools import product

 

SITES = {

        "ASHP": "careers.ashp.org",

        "APhA": "jobs.pharmacist.com",

        "ACCP": "careers.accp.com",

        "USAJOBS": "usajobs.gov",

        "Indeed": "indeed.com",

        "HigherEdJobs": "higheredjobs.com",

}

 

PHARMACY_TERMS = ["pharmacist", "PharmD", '"doctor of pharmacy"', "RPh"]

TECHNOLOGY_TERMS = [

        '"artificial intelligence"', '"generative AI"', '"machine learning"',

        '"large language model"', '"AI trainer"', '"AI evaluator"',

        "informatics", '"clinical decision support"', "automation",

        '"data analytics"', '"electronic health record"', '"digital health"',

        '"system validation"', "governance",

]

DATE_START = "2023-01-01"

DATE_END = "2026-08-10"

 

print("source\tquery\tdate_start\tdate_end")

for (source, domain), (pharmacy, technology) in product(

        SITES.items(), product(PHARMACY_TERMS, TECHNOLOGY_TERMS)

):

        query = f"site:{domain} {pharmacy} {technology}"

        print(f"{source}\t{query}\t{DATE_START}\t{DATE_END}")

 

### 2.6. Eligibility Criteria

Records were eligible when they (1) were U.S.-located, U.S.-remote, or explicitly accepted U.S. workers; (2) required or accepted a pharmacy degree, pharmacist licensure, pharmacist experience, or pharmacist-specific identity; (3) contained an AI-explicit duty or a substantive AI-enabling duty; and (4) provided sufficient retrievable text for coding. Exclusions comprised non-U.S. advertisements, news or educational pages, pharmaceutical-industry roles without pharmacist eligibility, advertisements in which AI appeared only in recruitment boilerplate, duplicate renderings of an included requisition, and records lacking adequate surviving evidence. Pharmacist-eligible but nonexclusive roles were retained with a scope flag for sensitivity interpretation.

### 2.7. Candidate Identification, Deduplication, and Verification

Candidates entered a discovery table before eligibility was presumed. Multiple URLs were consolidated when employer, title, location, and requisition number identified the same opportunity. National talent pools reproduced across city or state pages were counted once. Near-identical employer templates were flagged to prevent boilerplate from inflating competency frequencies. Each candidate underwent a row-level audit recording employer, title, source, location, requisition number when available, displayed posting or closing date, retrieval date, pharmacist-qualification evidence, AI or technology evidence, compensation when reported, page status, canonical URL, evidence grade, disposition, and rationale.

### 2.8. Evidence Grading and Date Handling

Grade A1 denoted an AI-explicit original employer, university, recruiter, platform, or government page; A2 denoted an AI-explicit record supported by a professional board, full-text job service, or concordant indexed copies when the original page was unavailable. B1 and B2 applied the same distinction to AI-enabling comparator records. Dynamic pages returning an empty shell were corroborated through employer search pages, requisition searches, professional boards, or concordant indexed records. Exact dates were transcribed only when displayed. Relative dates were preserved as relative and were not converted into calendar dates. Missing dates were coded ‘not reported’ with the retrieval date. Closed advertisements could remain eligible when adequate employer-originated or concordant full-text evidence survived.

### 2.9. AI-Assisted Screening and Human Verification

OpenAI ChatGPT/Codex in ChatGPT Work mode was used for query expansion, web discovery support, structured extraction, duplicate suggestions, preliminary coding, competency synthesis, and manuscript drafting. Short source-grounded evidence was extracted before summarization, and direct advertisement language was distinguished from analytic inference. The sole investigator reviewed inclusion decisions, URLs, dates, pharmacist eligibility, evidence excerpts, deduplication, and final competency assignments. AI-generated summaries were never accepted as evidence. Unsupported claims were removed and corrections retained; for example, a federal pain and opioid-safety position initially labeled as informatics was re-reviewed and excluded.

### 2.10. Inductive Coding and Competency Derivation

Competency derivation proceeded through three documented analytic levels. First, advertisement text was coded descriptively using short, task-proximal labels (for example, ‘compare paired outputs’, ‘verify claims, ‘construct clinical cases,’ and ‘implement AI-enabled pharmacokinetic/pharmacodynamic (PK/PD) use cases’). Second, codes were compared across records and grouped into 11 workforce-activity domains when they represented a common occupational function. One advertisement could support multiple domains, but every assignment required a retained source excerpt. Third, the 11 domains were conceptually consolidated into five higher-order AI workforce-readiness competencies by asking what AI-specific capability enabled the observed activity: understanding, evaluating, testing, improving, implementing, or governing an AI system. At this stage, established pharmacist expertise—pharmacotherapy, medication safety, evidence appraisal, calculations, PK/PD, and patient-specific judgment—was separated from the AI-specific capability through which that expertise was applied. The resulting evidence chain was therefore advertisement text → descriptive task code → workforce-activity domain → required pharmacist foundation → AI workforce-readiness competency → illustrative educational performance. Direct requirements, analytic interpretations, and educational recommendations remained separately labeled. This inductive progression is consistent with conventional qualitative content analysis, in which coding categories are derived from the textual data rather than imposed a priori [16].

### 2.11. Researcher Positioning, Reflexivity, and Trustworthiness

The sole investigator is a pharmacology professor and pharmacy educator with experience in PharmD curriculum and competency development, accreditation, assessment, institutional effectiveness, faculty development, and AI-supported educational scholarship. This expertise supported interpretation of pharmacist qualifications and educational implications but also created a potential expectancy bias toward finding curricular relevance. Reflexive safeguards therefore prioritized task-proximal coding, explicit separation of employer language from interpretation, retention of exclusions and contradictory cases, and avoidance of treating conventional informatics work as direct evidence of AI demand.

A retrospective trustworthiness analysis was conducted using Lincoln and Guba’s criteria of credibility, dependability, confirmability, and transferability [17], applied to the preparation, organization, and reporting phases of qualitative content analysis [18]. The analysis examined source verification and disconfirming cases; consistency and documentation of analytic decisions; traceability of interpretations to advertisement-level evidence; and the contextual information needed to judge applicability beyond the retrieved sample. The procedures and corresponding documentary evidence are summarized in Table 1.

**Table 1:** Application of Lincoln and Guba’s trustworthiness criteria in this study.

Criterion	Analytic Question	Procedures Used in This Study	Documentary Evidence
Credibility	Are the interpretations adequately grounded in the retrieved advertisements?	Original-source preference; pharmacist-eligibility verification; retained task excerpts; exclusion of unsupported records; disconfirming-case and sensitivity analyses	Advertisement-level evidence table; exclusion log; restrictive-set results
Dependability	Is the analytic process documented and consistently applied?	Explicit eligibility, deduplication, evidence-grading, coding, and scope rules; reproducible query generator; versioned corrections; locked dataset	Protocol; query code; candidate audit; dataset version 0.5
Confirmability	Can interpretations be traced to documentary evidence rather than assertion alone?	Evidence chain from advertisement text to descriptive code, workforce-activity domain, pharmacist foundation, and AI competency; separation of source language from interpretation; AI summaries not treated as evidence	Source excerpts; employer-level support matrix; competency-derivation table
Transferability	Is sufficient context provided for readers to judge applicability to other pharmacy settings?	Reporting of employer context, pharmacist scope, evidence grade, source type, work arrangement, and sample boundaries	Table 2; sector analysis; scope flags; limitations

**Table 2:** AI-explicit employment advertisements in the locked primary analytic sample.

Employer	Position	Date Evidence	Employment Context and Pharmacist Scope	Observed AI-Related Work
AuraOne	Clinical Pharmacist AI Evaluator	Date NR; retrieved 7 Aug 2026	External AI-evaluation platform; pharmacist-specific	Evaluate clinical reasoning; check dosing, safety and guidelines; document corrections; apply rubrics; calibration/drift
OpenTrain	Pharmacist AI Evaluation SME	9 Jul 2026	External AI-training platform; pharmacist-specific	Detect dosing, interaction, contraindication, monitoring and counseling errors; gold standards; prompts; biased/unsafe output
OpenTrain	Pharmacology AI Training Expert	21 Jul 2026	External AI-training platform; PharmD-eligible	Evaluate PK/PD, interactions and therapeutic use; construct cases and datasets; structured feedback
UNT Health	Assistant/Associate Professor	11 Jul 2026	Academic pharmacy; PharmD and licensure eligibility	Applied AI/digital health/CDS; responsible-use teaching; ethics, governance and patient-care integration
RYZ Labs client	Pharmacist–AI Trainer	Date NR; retrieved 9 Aug 2026	External AI recruitment/training platform; pharmacist-specific	Review prescription, interaction and education content; correct errors; create training sets; privacy and safeguards
DataAnnotation	Clinical Pharmacist–AI Trainer	Relative date; retrieved 10 Aug 2026	External AI-training platform; clinical pharmacist	Provide complex healthcare problems; evaluate logic, accuracy and performance; ensure medical accuracy
Prolific	Pharmacists–AI Training	One mirror: 1 Apr 2026	External AI research/training platform; licensed pharmacist	Evaluate accuracy, safety and reasoning; compare outputs; justify preferred answer; write exemplars and feedback
micro1	Clinical Pharmacist AI Trainer	Relative date; retrieved 10 Aug 2026	External AI-training platform; clinical pharmacist	Train/validate/review AI; analyze cases; evaluate output; iterative feedback; create datasets, guidelines and protocols
Taskify AI	Healthcare Expert (Remote)	Relative date; retrieved 10 Aug 2026	External AI-training platform; PharmD-eligible	Write/refine prompts; evaluate reasoning and completeness; verify claims; score models using frameworks/taxonomies
Novartis	Clinical Pharmacology AI Lead	26 Jun 2026	Pharmaceutical industry; PharmD-eligible	Lead AI-enabled clinical-pharmacology strategy; implement PK/PD, dosing, regulatory and automation use cases

### 2.12. Descriptive and Sensitivity Analyses

Counts were calculated at the distinct-requisition level. Competency frequencies used the 10 AI-explicit records; the 21 AI-enabling records were analyzed only as comparators. One advertisement could support multiple domains, making frequencies nonexclusive. Because the purposively retrieved corpus was small, frequencies were interpreted as workforce signals rather than national prevalence estimates. No post-2023 growth analysis was conducted because differential webpage attrition precluded valid longitudinal inference. A robustness analysis tested the stability of the five-competency synthesis under three progressively restrictive analytic sets: exclusion of the three A2 records lacking a directly accessible original employer page; exclusion of the three pharmacist-eligible but nonexclusive records retained with scope flags; and simultaneous restriction to A1 evidence and positions without scope flags. A competency was considered retained when at least one remaining advertisement contained a traceable task or requirement supporting the underlying workforce-activity domain. The analysis also examined disconfirming evidence: AI-enabling advertisements that did not explicitly require AI work, the absence of conventional community or hospital AI-explicit vacancies, and competencies supported by only a small minority of primary records.

### 2.13. Dataset Lock, Ethics, and Reproducibility

The dataset was locked on 10 August 2026 as version 0.5. It contained 34 assessed requisitions: 31 included records (10 AI-explicit primary advertisements and 21 AI-enabling comparators) and three exclusions. Versioned materials included the protocol, query inventory, executable code, search-log template, extraction template, audit workbook, analytic tables, and study-flow figure. The study analyzed public organizational documents and involved no human-participant interaction or identifiable private information; institutional review board review was considered not applicable, subject to confirmation under the investigator’s institutional policy.

## 3. Results

### 3.1. Identification and Classification of Advertisements

**Figure 1:**
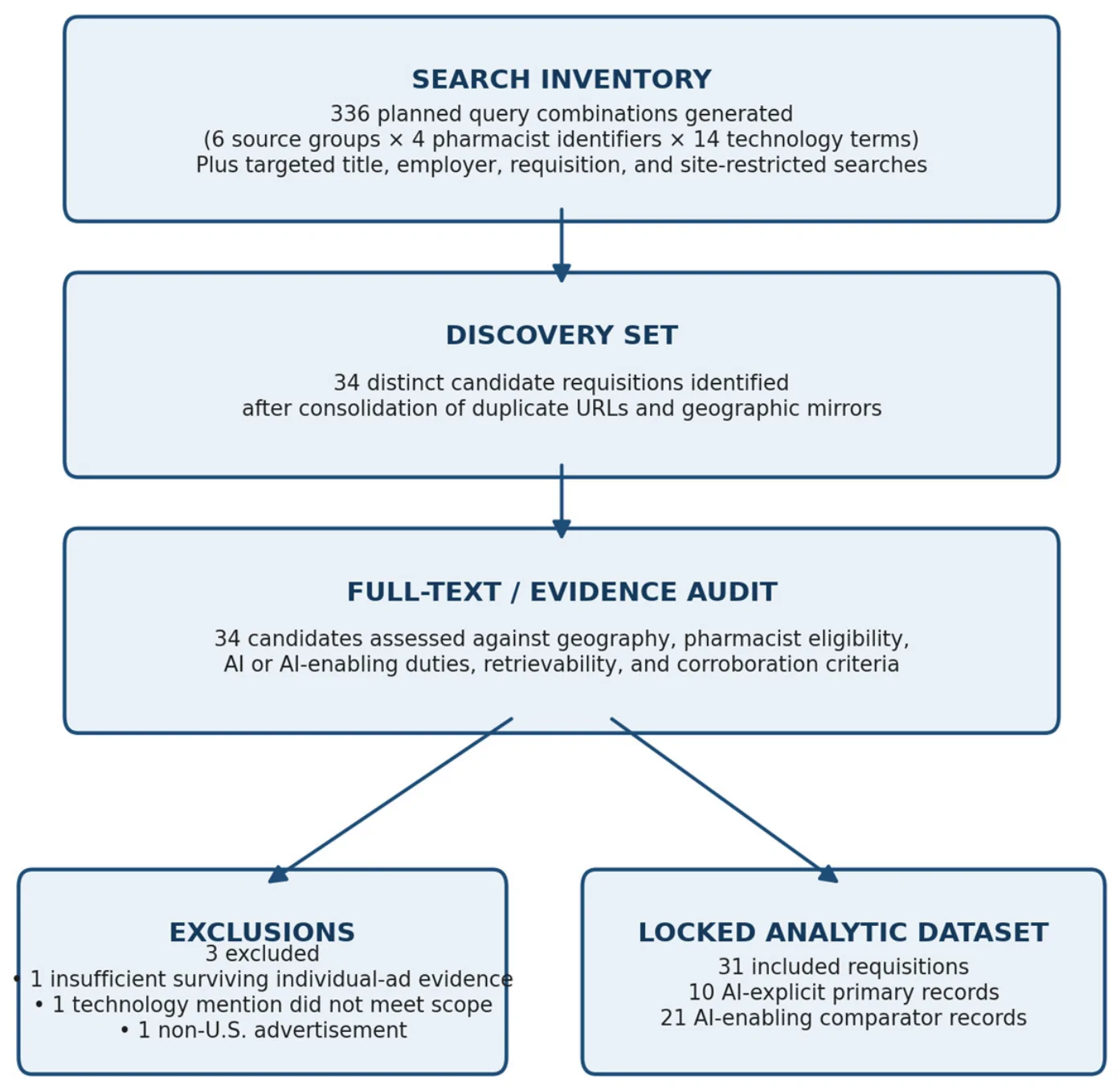
**Study Flow.** Identification, verification, and classification of pharmacist employment advertisements. Counts refer to distinct requisitions; duplicate URLs and duplicate location-specific postings were consolidated.

The audit assessed 34 distinct candidate requisitions after duplicate URLs and duplicate location-specific postings were consolidated. Thirty-one met the operational criteria, and three were excluded: one lacked adequate surviving individual-ad evidence, one technology-related pharmacist position did not meet the analytic scope after full review, and one advertisement was non-U.S. despite conflicting location metadata. Ten included records explicitly described AI-related work and formed the primary sample. The remaining 21 described informatics, EHR, automation, analytics, CDS, or technology implementation without an explicit AI duty and were retained only as contextual comparators (Figure 1).

### 3.2. Settings and Forms of AI-Explicit Work

The 10 AI-explicit records represented three employment contexts. Eight advertisements (80%) originated from external AI evaluation, training, expert-network, or recruitment platforms; one (10%) was an academic pharmacy faculty position; and one (10%) was a pharmaceutical-industry clinical-pharmacology implementation position. No AI-explicit advertisement in the primary sample was a conventional community-pharmacist or hospital/health-system pharmacist vacancy. These proportions characterize the retrieved pilot sample and should not be interpreted as national sector prevalence. Across these contexts, two emerging employment pathways were apparent. The first involved pharmacists as clinical evaluators, trainers, and quality reviewers for AI systems. AuraOne, OpenTrain, RYZ Labs, DataAnnotation, micro1, Prolific, and Taskify AI sought medication or healthcare expertise for evaluating model outputs, correcting reasoning, constructing cases or prompts, creating training materials, applying ratings, or providing feedback. Several were contractor, expert-network, project-intake, or talent-pool opportunities rather than conventional permanent pharmacist positions. The second pathway involved implementation of AI within established academic or pharmaceutical work. UNT Health sought faculty expertise spanning applied AI, digital health, clinical decision support, ethics, governance, and curricular integration. Novartis sought a PharmD-eligible clinical-pharmacology AI leader to develop and implement AI-supported PK/PD, dose-selection, automation, and regulatory use cases.

### 3.3. Pharmacists as Evaluators and Correctors of AI Reasoning

The dominant task was determining whether AI-generated clinical content was accurate, complete, logical, and safe. Eight of 10 advertisements supported evaluation of model output or reasoning, while seven directly supported clinical-accuracy or medication-safety validation. Employers asked pharmacists to identify dosing, interaction, contraindication, monitoring, counseling, or therapeutic errors; compare competing responses; justify the preferable answer; and supply corrected reasoning or structured feedback. Pharmacist expertise therefore served as a reference standard against which model performance was judged. The role was not simply to approve or reject an answer, but to externalize why it was acceptable, incomplete, or unsafe.

### 3.4. Clinical-Reasoning Codification and Reference Standards

Requests for gold-standard answers, exemplars, rationales, guidelines, protocols, and structured feedback indicated a need to codify clinical reasoning. A defensible reference answer must identify relevant patient variables, state a recommendation, explain its rationale, address alternatives and uncertainty, specify monitoring and safety contingencies, and distinguish unsafe reasoning from acceptable variation. The inferred competency was clinical-reasoning codification: translating pharmacotherapy expertise into explicit, patient-specific, machine-evaluable logic. Comparative output adjudication extended this competency by requiring pharmacists to select the stronger of two plausible responses and explain the clinically consequential difference.

### 3.5. Prompt, Case, Dataset, and Evaluation-Task Design

Seven advertisements supported the construction or refinement of prompts, complex healthcare problems, clinical cases, simulated data, datasets, training examples, protocols, or exemplars. These duties did not establish a universal requirement for programming or technical model development. Instead, they described clinical evaluation-task design. Pharmacists were expected to construct cases that revealed whether a model recognized organ dysfunction, pregnancy, age, concurrent drugs, laboratory findings, contraindications, missing information, or other variables that change medication decisions. Designing cases to surface unsafe or biased output was interpreted as adversarial medication-safety testing. The additional advertisements also expanded this domain into clinical dataset and protocol curation: maintaining the accuracy, representativeness, evidence base, and intended use of the materials through which AI is trained or assessed.

### 3.6. Structured Evaluation and Model-Performance Measurement

Six advertisements supported structured scoring, comparison, calibration, model-progress measurement, standardized frameworks, taxonomies, or iterative feedback. This transformed evaluation from an isolated fact check into a quality-management process. Pharmacists may need to define or apply criteria for accuracy, completeness, evidence alignment, severity of potential harm, uncertainty, and audience appropriateness; document disagreements; and recognize recurring errors across cases or model versions. DataAnnotation’s emphasis on measuring progress, Prolific’s paired-response comparison, micro1’s iterative feedback, and Taskify AI’s standardized scoring frameworks collectively supported a distinct competency in reproducible model-performance evaluation.

### 3.7. Quantitative Pharmacotherapy and Clinical-Pharmacology Applications

Seven advertisements implicated dosing, calculations, PK/PD, interactions, medication optimization, or clinical pharmacology. In the evaluator pathway, pharmacists checked model calculations, assumptions, units, and clinical interpretation. The Novartis role broadened the signal from validation to implementation by seeking AI-enabled approaches for PK/PD, dose selection, clinical-pharmacology strategy, regulatory deliverables, and automation. The resulting competency is not blind delegation of quantitative work. It is the ability to combine pharmacokinetic and pharmacodynamic expertise with AI-enabled analysis while independently verifying inputs, equations, assumptions, units, plausibility, patient applicability, and downstream decisions.

### 3.8. Evidence Verification, Bias, Governance, and Implementation

Evidence verification emerged as a distinct domain because employers requested comparison with guidelines, protocols, standards of care, and authoritative sources. The pharmacist must identify claims, locate appropriate evidence, determine whether it supports the response, assess currency and patient applicability, and document discrepancies. Bias, ethics, privacy, regulation, or governance appeared less consistently and were not always operationally defined. The directly supported signal was the ability to recognize and flag unsafe or potentially biased output; pharmacy-specific fairness assessment across age, sex and gender, pregnancy, disability, language, access, organ function, pharmacogenomics, and pharmacokinetic context remained an evidence-anchored inference. Novartis and UNT Health added a separate implementation competency: identifying appropriate AI use cases, integrating them with professional workflows and education, and establishing oversight and accountability.

### 3.9. From Workforce Activities to AI Workforce-Readiness Competencies

**Figure 2:**
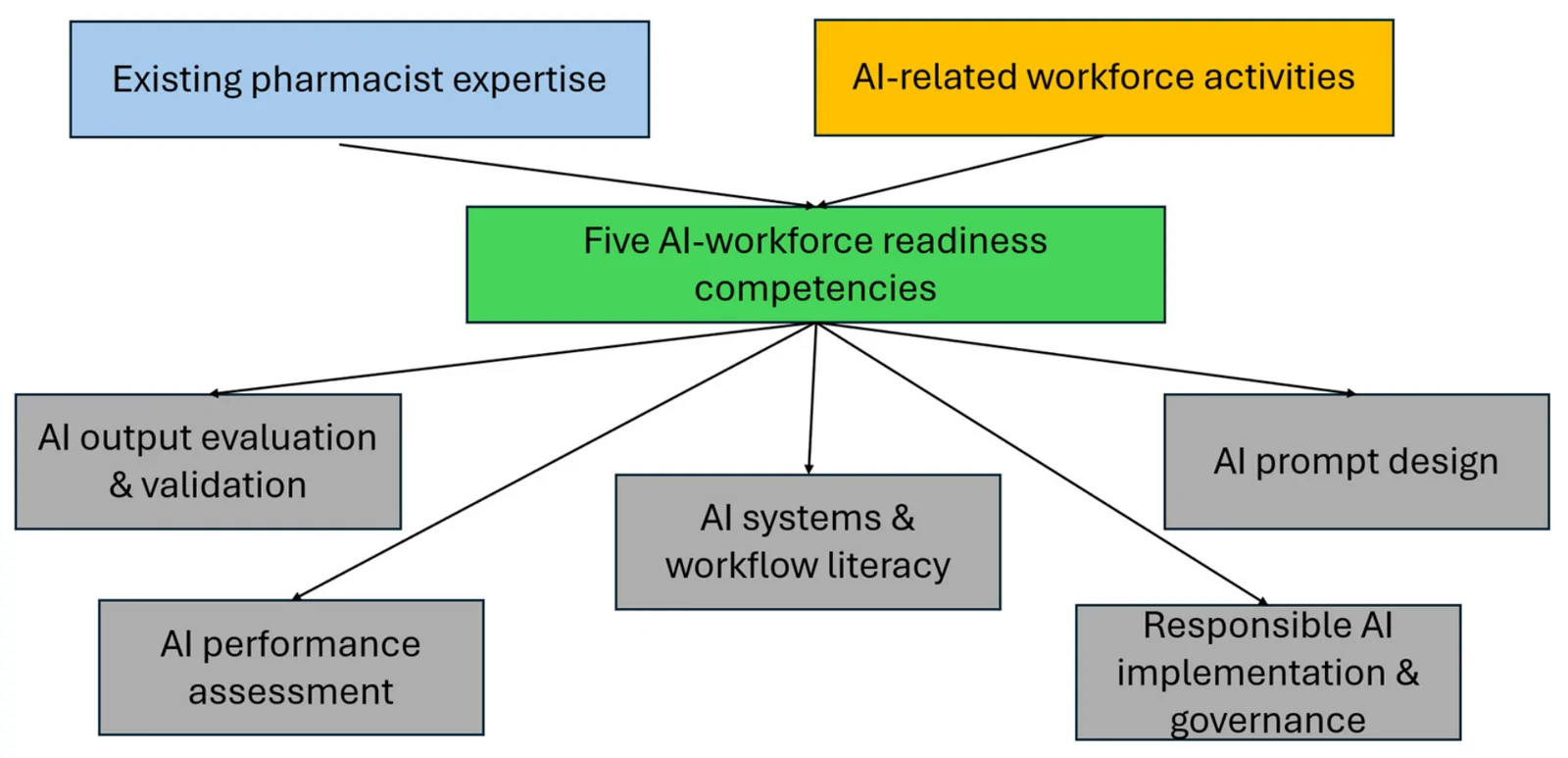
Derivation of five AI workforce-readiness competencies from the intersection of established pharmacist expertise and AI-related workforce activities observed in employment advertisements.

The 11 observed workforce-activity domains were synthesized into five AI workforce-readiness competencies (Table 3; Figure 2). AI-output evaluation and validation concerns judging and acting on a particular AI response. AI systems and workflow literacy concerns tracing how inputs, models, retrieval sources, tools, and human controls produced that response and where failure may occur. Prompt and evaluation-task design concerns constructing instructions, cases, reference standards, and tests that elicit useful performance or expose failure. AI performance assessment and improvement concerns reproducible evaluation across cases, populations, reviewers, and model versions. Responsible AI implementation and governance concerns selecting appropriate use cases and establishing workflow, privacy, fairness, safety, monitoring, escalation, and accountability controls. Pharmacotherapy, medication safety, evidence appraisal, calculations, and PK/PD were treated as the professional foundation needed to exercise these five AI-specific competencies—not as AI competencies themselves.

### 3.10. Robustness and Disconfirming-Case Analysis

All five higher-order competencies remained identifiable after removing the three A2 records (*n* = 7), after removing the three pharmacist-eligible but nonexclusive records (*n* = 7), and in the most restrictive intersection containing only A1 records without scope flags (*n* = 5). The breadth of support was not uniform. Output evaluation, prompt/evaluation-task design, and performance assessment were supported by multiple records in the restrictive set. Systems/workflow literacy and responsible implementation/governance remained identifiable principally through the academic position, with privacy and safeguard requirements providing additional governance evidence; they should therefore be regarded as lower-frequency, less-saturated competencies. The 21 comparator advertisements constituted an important negative case: they demonstrated substantial informatics, EHR, automation, analytics, and CDS demand without explicit AI duties and were not allowed to inflate the AI-derived framework. A second disconfirming pattern was sectoral: no conventional community or hospital/health-system pharmacist vacancy entered the AI-explicit primary set. The audit therefore supported the structural stability of the five-competency synthesis while narrowing the strength of claims about saturation, prevalence, and sectoral diffusion.

## 4. Discussion

### 4.1. Principal Findings

This targeted pilot environmental scan identified a small, purposively retrieved, and differentiated set of advertisements seeking pharmacist knowledge in AI. The expanded sample did not simply reproduce the original evaluator role. It revealed two related pathways: organizations purchasing pharmacist expertise to make AI-generated clinical reasoning trustworthy, and conventional institutions purchasing PharmD-compatible expertise to implement AI within education, patient care, clinical pharmacology, and drug development. The first pathway makes pharmacists external reference standards for AI quality. The second places them inside the design and operationalization of AI-enabled workflows. Together, they suggest task transformation rather than a single new job category. Importantly, the advertisements did not transform pharmacotherapy knowledge into an AI competency. They showed that existing professional expertise must be mobilized through five additional capabilities directed specifically toward AI systems: output validation, systems and workflow literacy, prompt and test design, performance assessment and improvement, and responsible implementation and governance. This redistribution of work is consistent with the broader conception of high-performance medicine as a human–AI partnership rather than simple technological substitution [19]. The documented strengths and limitations of large language models in medicine likewise reinforce the need for domain experts who can evaluate plausible outputs, identify consequential errors, and retain accountability for clinical decisions [20].

### 4.2. From Tacit Expertise to Machine-Evaluable Clinical Logic

The most consequential workforce signal was the conversion of tacit professional judgment into explicit artifacts: gold-standard answers, corrected rationales, prompts, cases, datasets, protocols, rating criteria, and escalation decisions. This work is epistemic rather than primarily computational. Employers need pharmacists to determine what counts as a defensible medication decision and to make the reasoning sufficiently explicit that it can train, test, or govern an AI system. This finding aligns with professional guidance assigning pharmacists roles in validation, scientific evaluation, oversight, and medication safety [1,2,3], while providing task-level examples of how those responsibilities may be operationalized.

### 4.3. Implications for Professional Roles and Automation

The advertisements demonstrate that current AI systems require expert review, yet pharmacists are simultaneously being recruited to improve those systems. This creates a potential automation paradox: the profession’s knowledge is needed to make AI more capable, including in tasks historically performed by pharmacists. The evidence cannot establish occupational replacement. A more defensible interpretation is redistribution of tasks. Retrieval, preliminary drafting, routine calculations, and first-pass reasoning may become more automated, while pharmacist responsibility shifts toward problem specification, validation, exception management, uncertainty, workflow integration, communication, and accountability. The profession’s strategic value may increasingly depend on its ability to codify and supervise medication reasoning rather than merely retain exclusive possession of drug information. These responsibilities align with international and national governance frameworks that emphasize human autonomy, safety, transparency, accountability, risk management, and continuing performance monitoring across the AI lifecycle [21,22,23].

### 4.4. Relationship Between AI-Explicit and AI-Enabling Work

The 21 comparator roles showed a much broader existing workforce concerned with medication data, EHR content, order sets, automation, interfaces, analytics, CDS, testing, implementation, and user support. These advertisements were not evidence that employers demanded AI competency, and preserving that boundary prevented inflation of the primary finding. They nevertheless identify the sociotechnical substrate through which AI output would reach prescribing, verification, dispensing, infusion, administration, and documentation. Safe AI therefore requires both model-focused competencies and established informatics capabilities. A clinically accurate model can remain unsafe when it receives incomplete data, appears at the wrong workflow point, conceals uncertainty, creates alert fatigue, or transmits an erroneous instruction downstream. Similar implementation analyses have emphasized that clinical AI depends on workflow integration, interoperable data, privacy protections, transparency, and patient-safety controls rather than algorithmic performance alone [24].

### 4.5. Implications for PharmD Education

Our preliminary workforce signals suggest five potential priorities for staged curricular development and subsequent validation.

Learners should be able to evaluate and validate AI outputs; explain how inputs, prompts, models, retrieval sources, embedded tools, and human oversight shape an output; design prompts and clinical evaluation tasks; assess performance across cases and model versions and provide structured improvement feedback; and determine whether and how an AI system should be implemented and governed (Table 4). These capabilities should be exercised through established pharmacist expertise in pharmacotherapy, medication safety, evidence appraisal, calculations, PK/PD, informatics, ethics, and patient-specific judgment. The empirical support was not equivalent across the framework: output evaluation, prompt and evaluation-task design, and performance assessment were supported by multiple records in the most restrictive sensitivity set, whereas systems/workflow literacy and responsible implementation/governance were less saturated and require further validation. They can be integrated across existing courses, skills laboratories, simulation, and experiential education rather than confined to a single elective (Table 3). This staged and competency-based approach is consistent with a scoping review of AI education for health professionals, which identified interpretation, explanation, implementation, regulation, and multidisciplinary curricular design as recurring educational priorities [25].

### 4.6. Prompt Competence as Clinical Test Design

Prompt competence should not be reduced to clever phrasing of chatbot requests. The observed work more closely resembles clinical test design: specifying the problem, relevant context, expected reasoning, acceptable answer boundaries, and failure criteria. Learners should be able to vary clinically consequential parameters, predict how a safe response should change, and recognize whether the model requests missing information. Existing pharmacy simulation work demonstrates that structured prompts can encode patient context, professional roles, and decision requirements [12]. The workforce evidence extends that approach by positioning pharmacists as designers of tasks that distinguish superficial pattern matching from clinically safe reasoning.

### 4.7. Quantitative Validation and Evidence Grounding

Quantitative and evidence-verification competencies are particularly suitable for observable assessment. Students can be required to audit inputs, units, equations, assumptions, outputs, and downstream actions in AI-assisted dosing or PK/PD problems. They can identify claims in model responses, retrieve authoritative evidence, determine whether citations are real and applicable, and document discrepancies. The Novartis advertisement further suggests that advanced or postgraduate preparation may incorporate AI-enabled clinical pharmacology, dose selection, and regulatory-science applications. These activities preserve the pharmacist’s responsibility to validate rather than ceremonially approve automated output.

### 4.8. Research and Validation Agenda

The proposed competencies remain workforce-anchored hypotheses rather than accreditation standards. The next phase should prospectively repeat and archive searches, obtain independent coding, interview hiring managers and incumbents, and seek consensus among practicing pharmacists, informaticists, regulators, educators, students, technology developers, and patients. Competencies should then be mapped to existing pharmacy educational outcomes and translated into observable performance indicators. Particular attention is required to distinguish durable professional functions from temporary model-training labor and to determine whether evaluation and governance remain pharmacist responsibilities as models mature.

## 5. Limitations

This study has limitations. First, the 10 AI-explicit advertisements constitute a small, purposively retrieved sample; frequencies are descriptive signals and not estimates of national prevalence. Second, open-web advertisements are transient. Older postings disappear, applicant-tracking systems restrict indexing, and some services display relative dates or incomplete descriptions. The intended 2023–2026 coverage window therefore cannot establish that AI-related pharmacist work increased after ChatGPT’s release. A valid trend analysis would require prospective repeated searches or a historical labor-market archive.

Third, the scripted matrix generated an inventory of 336 term-and-source combinations, but it does not establish that all 336 strings were executed, and a complete query-level execution log was not preserved; the study should therefore be interpreted as a targeted pilot environmental scan rather than an exhaustive or systematic search. Fourth, evidence accessibility varied. Original sources were preferred, but some records required professional-board, full-text job-service, or indexed corroboration. Several AI-explicit opportunities were contractor, project-intake, or talent-pool postings, and some accepted PharmD preparation without being pharmacist-exclusive. They demonstrate demand for pharmacy expertise but should not be equated with permanent pharmacist vacancies. Job advertisements express desired qualifications rather than verified daily practice, while employers may use AI without naming it in advertisements. Fifth, qualitative interpretation extended beyond literal wording when moving from observed workforce activities to higher-order AI workforce-readiness competencies. Traceable evidence chains were retained, but a second coder could organize the domains differently. Finally, generative AI supported searching, extraction, coding, and drafting, creating a risk of unsupported synthesis. Human verification, evidence grading, preserved corrections, and exclusion of unsupported records reduced but did not eliminate that risk. Employer interviews, dual coding, stakeholder consensus, and educational validation are required before the framework is considered comprehensive.

## 6. Conclusions

Within this targeted pilot environmental scan, publicly retrievable employment advertisements indicated two emerging uses of pharmacist expertise: evaluating and improving clinical AI systems and implementing AI-enabled medication-use, educational, clinical-pharmacology, and drug-development workflows. The findings distinguish the pharmacist’s established professional foundation from five AI-specific capabilities that may be relevant to an AI-mediated workplace. Three competencies—AI-output evaluation and validation, prompt and evaluation-task design, and AI performance assessment and improvement—were supported by multiple advertisements in the most restrictive sensitivity set. AI systems and workflow literacy and responsible AI implementation and governance were less saturated and should be treated as preliminary competencies requiring additional employer and stakeholder validation. Accordingly, the five-competency framework identifies potential priorities for pharmacy education rather than established curricular requirements. These priorities are to (1) evaluate and validate AI-generated outputs; (2) understand and trace the AI systems and workflows that produce them; (3) design prompts, cases, reference standards, and evaluation tasks; (4) assess performance across cases and model versions and provide structured feedback for improvement; and (5) implement and govern AI responsibly within pharmacy workflows. The framework provides a preliminary basis for prospective workforce research, employer validation, and development of observable educational outcomes.

## Figures and Tables

**Table 3:** Five AI workforce-readiness competencies derived from observed employment activities.

	AI Workforce-Readiness Competency	Definition	Observed Workforce Evidence	Required Pharmacist Foundation	Illustrative Educational Performance
1	AI-output evaluation and validation	Systematically determine whether an AI-generated pharmacy output is accurate, complete, clinically reasoned, appropriately uncertain, and safe; accept, correct, escalate, or reject it.	Output review; medication-safety validation; corrective reasoning; comparative adjudication; evidence and quantitative verification.	Pharmacotherapy, medication safety, calculations, PK/PD, evidence appraisal, and patient-specific clinical judgment.	Audit an AI-generated therapeutic recommendation, document consequential errors or omissions, and provide a defensible corrected response.
2	AI systems and workflow literacy	Explain and trace how inputs, prompts, data or retrieval sources, model behavior, tools, and human oversight combine to produce an AI output, including where failure may occur.	Recognition of hallucination, inconsistency, missing context, bias, data limitations, and the distinction between model output and verified clinical reasoning.	Medication-use processes, health informatics, clinical decision support, data provenance, and professional accountability.	Map an AI-supported medication workflow, identify failure points and missing controls, and explain why a plausible output may still be unsafe.
3	Prompt and evaluation-task design	Construct and refine prompts, cases, test scenarios, expected-answer criteria, and reference materials that elicit useful outputs and expose clinically consequential failure modes.	Prompt construction; clinical case and problem design; adversarial testing; dataset, protocol, exemplar, and gold-standard development.	Clinical problem formulation, patient-context specification, case construction, and articulation of acceptable therapeutic reasoning.	Design a prompt and case set in which clinically consequential variables are varied, with expected responses and explicit failure criteria.
4	AI performance assessment and improvement	Apply reproducible rubrics, benchmarks, comparative review, calibration, and repeated testing to characterize performance patterns and provide structured feedback for improvement.	Scoring frameworks and taxonomies; paired-output comparisons; error-pattern analysis; model-progress measurement; iterative feedback and reference-set curation.	Quality improvement, assessment design, evidence standards, severity-of-harm judgment, and interrater reasoning.	Use a defined rubric across multiple cases or model versions, analyze recurring errors and variability, and recommend evidence-grounded improvements.
5	Responsible AI implementation and governance	Determine whether AI is appropriate for a pharmacy problem; integrate it into a workflow with privacy, fairness, safety, monitoring, human review, escalation, and accountability controls.	AI use-case identification; clinical and educational workflow integration; ethics, privacy, bias, governance, regulation, monitoring, and clinical-to-technical translation.	Medication-use systems, implementation science, ethics, regulation, patient safety, communication, and organizational leadership.	Conduct a pre-implementation assessment defining intended use, evidence threshold, human authority, escalation rules, monitoring indicators, and stop criteria.

**Table 4:** Educational translation and assessment of the five AI workforce-readiness competencies.

Competency	Expected Performance	Suggested Level	Workforce Anchor
AI-output evaluation and validation	Audit an AI-generated therapeutic recommendation, document consequential errors or omissions, and provide a defensible corrected response.	Foundational	Output review; medication-safety validation; corrective reasoning; comparative adjudication; evidence and quantitative verification.
AI systems and workflow literacy	Map an AI-supported medication workflow, identify failure points and missing controls, and explain why a plausible output may still be unsafe.	Foundational	Recognition of hallucination, inconsistency, missing context, bias, data limitations, and the distinction between model output and verified clinical reasoning.
Prompt and evaluation-task design	Design a prompt and case set in which clinically consequential variables are varied, with expected responses and explicit failure criteria.	Developing/Applied	Prompt construction; clinical case and problem design; adversarial testing; dataset, protocol, exemplar, and gold-standard development.
AI performance assessment and improvement	Use a defined rubric across multiple cases or model versions, analyze recurring errors and variability, and recommend evidence-grounded improvements.	Developing/Applied	Scoring frameworks and taxonomies; paired-output comparisons; error-pattern analysis; model-progress measurement; iterative feedback and reference-set curation.
Responsible AI implementation and governance	Conduct a pre-implementation assessment defining intended use, evidence threshold, human authority, escalation rules, monitoring indicators, and stop criteria.	Advanced	AI use-case identification; clinical and educational workflow integration; ethics, privacy, bias, governance, regulation, monitoring, and clinical-to-technical translation.

## Data Availability

The advertisement-level audit data and derived competency framework are provided in Supplementary File S1. The workbook includes retrieval dates, canonical URLs, evidence grades, pharmacist-eligibility determinations, classification decisions, and short source-grounded descriptions. Because employment advertisements are dynamic and may expire, full advertisement text is not reproduced. The query-generation code and search templates are available from the author upon reasonable request.

## Supplementary Materials

The following supporting information can be downloaded at https://www.mdpi.com/article/10.3390/pharmacy14060138/s1: Supplementary File S1: Advertisement audit and AI workforce-readiness competency framework. The Excel workbook contains Table S1, AI-explicit advertisements included in the primary analysis; Table S2, AI-enabling comparator advertisements; Table S3, complete candidate-level verification audit and exclusion decisions; and Table S4, five AI workforce-readiness competencies derived from observed employment activities.
