# Supplementary material for: Distilling AI Workforce-Readiness Competencies for U.S. Pharmacists from Job Advertisements: Implications for Pharmacy Education
Source: Pharmacy (Basel). 2026 Sep 20;14(6):138. doi: 10.3390/pharmacy14060138 (PMC13610747; doi:10.3390/pharmacy14060138)
Supplement: Supplementary file 1 [file pharmacy-14-00138-s001.zip › pharmacy-4530365-supplementary.pdf]

Supplementary File S1A - Read Me

	Advertisement Audit and AI Workforce-Readiness Competency Framework
**Study title**	Distilling AI Workforce-Readiness Competencies for U.S. Pharmacists from Job Advertisements: Implications for Pharmacy Education
**Dataset lock**	10 August 2026; version 0.5
**Unit of analysis**	Distinct employment requisition. Duplicate URLs and duplicate location-specific postings representing the same opportunity were consolidated.
**Primary sample**	10 AI-explicit advertisements that directly described AI, generative AI, machine learning, model training, model evaluation, or AI-enabled implementation work.
**Comparator sample**	21 AI-enabling pharmacist advertisements involving informatics, EHRs, automation, analytics, clinical decision support, system validation, or digital governance without an explicit AI duty. These were not counted as evidence of direct AI competency demand.
**Complete audit**	34 candidate requisitions: 31 included and 3 excluded with reasons retained.
**Evidence grade A1**	AI-explicit record supported by an original employer, university, recruiter, platform, or government source.
**Evidence grade A2**	AI-explicit record supported by a professional board, full-text job service, or concordant indexed copies when an original page was unavailable.
**Evidence grades B1/B2**	The corresponding original-source or corroborated-secondary grades applied to AI-enabling comparator records.
**Date handling**	Exact dates were recorded only when displayed. Relative dates were preserved as relative; unavailable dates were coded as not reported with retrieval date retained.
**Workbook contents**	Table S1: 10 AI-explicit advertisements; Table S2: 21 AI-enabling comparators; Table S3: complete 34-record verification audit; Table S4: five AI workforce-readiness competencies.
**Source-text limitation**	Short evidence-grounded descriptions are provided. Full advertisement text is not reproduced because webpages are dynamic and may be subject to copyright or platform terms.

Supplementary Table S1. AI-Explicit Advertisements

ID	Audit decision	Evidence grade	Page status 2026-08-09	Posting date status	Credential evidence (short source excerpt)	Technology evidence (short source excerpt)	Audit finding	Clickable source
32	Include with scope flag	A2	LinkedIn full text; closed posting	Relative: 4–5 months; retrieved 2026-08-10	Advanced healthcare credential including PharmD; ≥5 years healthcare experience	Write/refine prompts; evaluate accuracy, reasoning and completeness; verify claims; score models using frameworks/taxonomies	U.S. candidates accepted, but role is healthcare-wide rather than pharmacist-exclusive; sensitivity flag retained.	https://www.linkedin.com/jobs/view/healthcare-expert-remote-at-taskify-ai-4379701904
1	Include	A1	Live primary/recruiter page	Not reported	Active or recent license / board certification in pharma or an adjacent specialty	Review AI outputs against current pharma clinical guidelines and standard of care	Pharmacist-specific AI-evaluation duties and credential language directly confirmed.	https://auraone.ai/public/ai-labs/jobs/clinical-pharmacist-ai-evaluator-aoj-curated-f66fe7dcfa5ca0e338
2	Include	A1	Live platform/employer page	Exact: 2026-07-09	PharmD or Bachelor's/Master's degree in Pharmacy with active pharmacist licensure or eligibility	Identify errors in dosing, interactions, contraindications, monitoring, and counseling	Date, U.S. eligibility, compensation, credentials, and AI duties directly confirmed.	https://www.opentrain.ai/jobs/pharmacist-ai-evaluation-sme--cmrd8x3ur001mnj3lgybb3ucm/
33	Include with scope flag	A1	Live original Novartis employer page	Exact: 2026-06-26	PhD or PharmD with pharmacokinetics/drug-metabolism background	Lead AI-enabled clinical-pharmacology strategy; develop AI use cases for PK/PD, dosing, regulatory deliverables and automation	Conventional pharmaceutical-industry role; pharmacist-eligible rather than pharmacist-exclusive.	https://www.novartis.com/careers/career-search/job/details/req-10081408-principal-scientist-iisenior-principal-scientist-pk-sciences-clinical-pharmacology-artificial-intelligence-ai-lead-dual-level-posting
4	Include with scope flag	A1	Live platform/employer page	Exact: 2026-07-21	PharmD/PhD/MD candidates	Train and evaluate AI responses about pharmacokinetics, pharmacodynamics, drug interactions, and therapeutic use	Pharmacists are eligible but the role is not pharmacist-exclusive; retain separately for sensitivity analysis.	https://www.opentrain.ai/jobs/pharmacology-ai-training-expert--cmrufsk8y000k04jfuq7bepii/
6	Include	A1	Live university employer page	Exact: 2026-07-11	PharmD and Texas pharmacist licensure eligibility	Applied AI, digital health, analytics, CDS, governance, ethics and curriculum integration	Fully corroborated through UNT employer page; high-value AI-explicit academic record.	https://careers.ashp.org/job/assistant-associate-professor-fort-worth-texas-14753
28	Include	A1	Live recruiter page	Not reported	Active pharmacist license in good standing	Analyze and review AI-generated content related to prescriptions, drug interactions, and patient education	Primary recruiter page directly confirms pharmacist licensure and detailed AI-training, safety, ethics, privacy, and communication duties.	https://www.ryzlabs.com/jobs/pharmacist-ai-trainer-remote-41d28
29	Include; geographic-mirror cluster collapsed	A2	LinkedIn full text plus concordant indexed copies	Relative date displayed; retrieved 2026-08-10	Existing clinical pharmacist; Medication Management (PharmD) expertise	Provide complex healthcare problems; evaluate AI logic, accuracy, and performance; ensure medical accuracy and response quality	One nationwide DataAnnotation opportunity; state mirrors collapsed; U.S.-only contractor.	https://www.linkedin.com/jobs/view/clinical-pharmacist-ai-trainer-at-dataannotation-4377553542
30	Include; geographic-mirror cluster collapsed	A1	Live original Greenhouse employer page plus indexed copies	Exact date available on one mirror: 2026-04-01; retrieved 2026-08-10	Licensed pharmacist/PharmD or RPh in good standing; recent practice experience	Evaluate pharmaceutical scenarios for accuracy, safety and reasoning; compare outputs; justify preferred answer; write exemplars and structured feedback	One Prolific expert-pool opportunity; numerous U.S. city mirrors collapsed.	https://job-boards.eu.greenhouse.io/prolificacademicltd/jobs/4869747101
31	Include	A2	LinkedIn full text; closed posting	Relative: 8 months; retrieved 2026-08-10	Clinical pharmacist; pharmacotherapy, medication-management and clinical-decision expertise	Train, validate and review AI; analyze cases/labs; evaluate outputs; give iterative feedback; create datasets, guidelines and protocols	Remote contract; North American version retained; duplicate micro1 title variants treated as one template cluster.	https://www.linkedin.com/jobs/view/clinical-pharmacist-ai-trainer-at-micro1-4340109569

Supplementary Table S2. AI-Enabling Comparator Advertisements

ID	Audit decision	Evidence grade	Page status 2026-08-09	Posting date status	Credential evidence (short source excerpt)	Technology evidence (short source excerpt)	Audit finding	Clickable source
5	Include	B1	Live employer page	Not reported on accessible page	Doctor of Pharmacy (PharmD) required; Registered Pharmacist required	Leveraging the power of artificial intelligence, automation, and digital technologies	Primary employer page confirms credentials and technology context; role duties emphasize EHR implementation and education.	https://careers.hcahealthcare.com/jobs/17995205-expanse-education-pharmacist
7	Include	B1	Closed but retrievable government page	Exact: 2026-07-24 to 2026-08-03	Pharmacy degree and pharmacist licensure required	Develops, designs, implements and manages a pharmacology informatics program	Primary government record is durable and directly supports inclusion.	https://www.usajobs.gov/job/877944900
8	Include	B1	Live government page	Exact: 2026-08-05 to 2026-08-18	Licensed pharmacist; ACPE pharmacy degree and unrestricted license	EHR, SQL, Power BI, dashboards, data analysis, integration, troubleshooting and informatics leadership	Fully corroborated government record.	https://www.usajobs.gov/job/879346500
9	Include	B1	Live government page	Exact: 2026-08-03 to 2026-08-17	0660 Pharmacist; licensure verification required	Automation, health-information systems, drug file/eMAR, testing, troubleshooting and technical guidance	Fully corroborated government record.	https://www.usajobs.gov/job/879020000
10	Include	B1	Live government page	Closed 2026-08-10; opening date NR in captured text	Pharmacist; full unrestricted pharmacy license	BCMA, CPRS, VistA, data analysis, system integration, optimization and actionable reporting	Fully corroborated government record; opening date remains NR rather than inferred.	https://www.usajobs.gov/job/879398100
11	Include	B1	Closed but fully retrievable government page	Exact: 2026-07-27 to 2026-08-05	Pharm.D./BS Pharmacy and unrestricted pharmacist license	Pharmacy information systems, data analytics, automation platforms, and technology-enabled workflows	Primary record directly confirms date, salary, credentials, duties, analytics, training, governance, and change management.	https://www.usajobs.gov/job/878124700
13	Include	B1	Employer Workday record indexed	Exact: 2026-06-19	Graduate of an ACPE-accredited School of Pharmacy with a Bachelor of Science in Pharmacy degree or higher	Business intelligence, analytics, and pharmacy informatics	Date, pharmacy education, title and technology focus corroborated through the employer record.	https://uasys.wd5.myworkdayjobs.com/en-US/UASYS/job/Pharmacy-Informatics-Analyst_R0087138-1
14	Include; template sensitivity flag	B1	Employer Workday record indexed	Exact date NR; retrieved 2026-08-09	Clinical pharmacist requisition	EHRs, pharmacy informatics systems, medication automation and CDS optimization	Genuine employer record; standardized AdventHealth competency language counted once in template-collapsed analysis.	https://adventhealth.wd12.myworkdayjobs.com/en-US/AH_External_Career_Site/job/Clinical-Pharmacist-Nonexempt_R-0369605-1
15	Include; template sensitivity flag	B1	Employer Workday record indexed	Relative date displayed; exact date NR; retrieved 2026-08-09	Pharmacist requisition	EHR/pharmacy informatics, medication automation and clinical decision support	Genuine employer record; standardized competency language flagged for template collapse.	https://adventhealth.wd12.myworkdayjobs.com/en-US/AH_External_Career_Site/job/Pharmacist_R-0343323-1
16	Include	B1	Employer Workday record indexed	Exact: 2026-07-29	Inpatient Clinical Pharmacist requisition	EHRs, pharmacy informatics systems, and medication automation	Exact date, role identity and technology competency are corroborated; shared-template sensitivity flag applies.	https://adventhealth.wd12.myworkdayjobs.com/ah_external_career_site/job/adventhealth-la-grange-mem/inpatient-clinical-pharmacist_r-0385587
17	Include; template sensitivity flag	B1	Employer Workday record indexed	Exact date NR; retrieved 2026-08-09	Clinical Pharmacist Part-Time requisition	EHRs, pharmacy informatics systems, medication automation and CDS	Genuine employer record; standardized competency language flagged for template collapse.	https://adventhealth.wd12.myworkdayjobs.com/en-US/AH_External_Career_Site/job/Clinical-Pharmacist--Part-Time_R-0377061
18	Include; template sensitivity flag	B2	ACCP professional job-board record	Exact date NR; retrieved 2026-08-09	Advanced specialty clinical pharmacist role	EHR/informatics, automation, CDS, medication safety and teaching	Professional-board evidence is adequate for inclusion but employer/date corroboration is limited; analyze separately from B1.	https://careers.accp.com/job/advanced-heart-failure-cardiac-transplant-clinical-pharmacist-orlando-florida-70050
19	Include	B1	Employer Workday record and independent full-text mirror corroborated	Reposted in 2026; exact original date pending	Graduate of an accredited College of Pharmacy; Kentucky/Indiana license or eligibility	IT, automation systems, medication-use process, implementation, integration, training and system changes	Credentials and detailed duties corroborated; exact original posting date remains NR rather than inferred.	https://bhs.wd1.myworkdayjobs.com/en-US/careers/job/System-Informatics-Pharmacist-Specialist---Remote-from-IN-or-KY_R26099483
20	Include	B1	Employer Workday record indexed	Exact: 2026-07-29	Clinical Informatics Pharmacist requisition	Primary subject-matter expert for pharmacy clinical information systems	Employer, requisition, exact date, pharmacist identity and informatics function corroborated.	https://umiami.wd1.myworkdayjobs.com/en-US/UMCareerStaff/job/Clinical-Pharmacist---IT-Systems---Full-Time_R100098946
21	Include	B1	Live employer career page	Exact date not displayed in captured text	Informatics Pharmacist role; licensure details require expanded capture	IT components connected to ordering, dispensing, or administration of medications	Employer page confirms requisition R-10977, duties, location, and $158,288-$237,432 salary.	https://www.overlakecareers.org/job/informatics-pharmacist-clinical-specialist-pharmacy-us-wa-bellevue-1035-116th-ave-ne-building-r-10977-49/
22	Include	B1	Accessible employer-hosted career page	Current in August 2026; exact date not displayed	Pharmacist; Louisiana Board of Pharmacy licensure corroborated	Build, implementation, integration and maintenance of automation and technology	Employer page confirms the role and duties; date is recorded as NR/retrieved rather than inferred.	https://lcmchealth.wd1.myworkdayjobs.com/LCMCHealth/job/LCMC-Health-Corporate/XMLNAME-340B-Clinical-Informatics-Pharmacist_JR0039574-1
23	Include	B1	Employer Workday record indexed	Current August 2026; exact date not captured	Current Indiana Pharmacist License or eligibility	Clinical pharmacy informatics coordination	Employer, requisition, licensure and informatics responsibility corroborated; exact date remains NR.	https://bhs.wd1.myworkdayjobs.com/careers/job/New-Albany-IN/Pharmacy-Informatics-Coordinator_R26104886
24	Include	B1	Employer Workday record plus independent indexed copy	Exact date NR; retrieved 2026-08-09	Clinical Informatics Pharmacist requisition	Clinical leadership for complex implementation; pharmacy informatics, automation and controlled-substance support	Employer requisition R00047937 and job function corroborated; exact posting date remains NR.	https://ummc.wd5.myworkdayjobs.com/en-US/umccareers/job/jackson-ms---main-campus/clinical-informatics-pharmacist---automation-and-narcotic-support---full-time_r00047937-2
25	Include	B1	Employer Oracle record plus independent full-text indexes	Exact: 2026-01-13	Clinical Informatics Pharmacist Specialist	Clinical drug and medical-device data translated into decision-support knowledge	Employer, requisition, title, date and informatics function corroborated.	https://eevd.fa.us6.oraclecloud.com/hcmUI/CandidateExperience/en/sites/FDB/job/2025396
26	Include	B1	Closed employer record/archived text corroborated	Closed: 2026-05-14	Clinical Informatics Pharmacist role	EHR medication content, order sets, CDS, workflow, testing, validation and implementation	Detailed employer-originated duties, compensation and closing date are available; opening date remains NR.	https://imh.wd108.myworkdayjobs.com/en-US/IntermountainCareers/job/Clinical-Informatics-Pharmacist_R170123
27	Include	B1	Employer page indexed	Exact: 2026-05-18	PharmD/equivalent and active pharmacist license	Epic configuration, smart infusion-pump integration, system build and informatics leadership	Employer, date, credentials and core duties corroborated.	https://careers.mountsinai.org/jobs/3038168?lang=en-us

Supplementary Table S3. Complete Candidate-Level Verification Audit

ID	Audit decision	Evidence grade	Page status 2026-08-09	Posting date status	Credential evidence (short source excerpt)	Technology evidence (short source excerpt)	Audit finding	Clickable source
1	Include	A1	Live primary/recruiter page	Not reported	Active or recent license / board certification in pharma or an adjacent specialty	Review AI outputs against current pharma clinical guidelines and standard of care	Pharmacist-specific AI-evaluation duties and credential language directly confirmed.	https://auraone.ai/public/ai-labs/jobs/clinical-pharmacist-ai-evaluator-aoj-curated-f66fe7dcfa5ca0e338
2	Include	A1	Live platform/employer page	Exact: 2026-07-09	PharmD or Bachelor's/Master's degree in Pharmacy with active pharmacist licensure or eligibility	Identify errors in dosing, interactions, contraindications, monitoring, and counseling	Date, U.S. eligibility, compensation, credentials, and AI duties directly confirmed.	https://www.opentrain.ai/jobs/pharmacist-ai-evaluation-sme--cmrd8x3ur001mnj3lgybb3ucm/
3	Exclude from primary; retain sensitivity	A2	Category page live; individual advertisement removed	Reported 2026-07-25 on category page	Active U.S. pharmacist license reported	AI review reported; individual full text unavailable	Cannot support full primary-source extraction; retained only in excluded/sensitivity log.	https://www.opentrain.ai/ai-training-careers/pharmacy/
4	Include with scope flag	A1	Live platform/employer page	Exact: 2026-07-21	PharmD/PhD/MD candidates	Train and evaluate AI responses about pharmacokinetics, pharmacodynamics, drug interactions, and therapeutic use	Pharmacists are eligible but the role is not pharmacist-exclusive; retain separately for sensitivity analysis.	https://www.opentrain.ai/jobs/pharmacology-ai-training-expert--cmrufsk8y000k04jfuq7bepii/
5	Include	B1	Live employer page	Not reported on accessible page	Doctor of Pharmacy (PharmD) required; Registered Pharmacist required	Leveraging the power of artificial intelligence, automation, and digital technologies	Primary employer page confirms credentials and technology context; role duties emphasize EHR implementation and education.	https://careers.hcahealthcare.com/jobs/17995205-expanse-education-pharmacist
6	Include	A1	Live university employer page	Exact: 2026-07-11	PharmD and Texas pharmacist licensure eligibility	Applied AI, digital health, analytics, CDS, governance, ethics and curriculum integration	Fully corroborated through UNT employer page; high-value AI-explicit academic record.	https://careers.ashp.org/job/assistant-associate-professor-fort-worth-texas-14753
7	Include	B1	Closed but retrievable government page	Exact: 2026-07-24 to 2026-08-03	Pharmacy degree and pharmacist licensure required	Develops, designs, implements and manages a pharmacology informatics program	Primary government record is durable and directly supports inclusion.	https://www.usajobs.gov/job/877944900
8	Include	B1	Live government page	Exact: 2026-08-05 to 2026-08-18	Licensed pharmacist; ACPE pharmacy degree and unrestricted license	EHR, SQL, Power BI, dashboards, data analysis, integration, troubleshooting and informatics leadership	Fully corroborated government record.	https://www.usajobs.gov/job/879346500
9	Include	B1	Live government page	Exact: 2026-08-03 to 2026-08-17	0660 Pharmacist; licensure verification required	Automation, health-information systems, drug file/eMAR, testing, troubleshooting and technical guidance	Fully corroborated government record.	https://www.usajobs.gov/job/879020000
10	Include	B1	Live government page	Closed 2026-08-10; opening date NR in captured text	Pharmacist; full unrestricted pharmacy license	BCMA, CPRS, VistA, data analysis, system integration, optimization and actionable reporting	Fully corroborated government record; opening date remains NR rather than inferred.	https://www.usajobs.gov/job/879398100
11	Include	B1	Closed but fully retrievable government page	Exact: 2026-07-27 to 2026-08-05	Pharm.D./BS Pharmacy and unrestricted pharmacist license	Pharmacy information systems, data analytics, automation platforms, and technology-enabled workflows	Primary record directly confirms date, salary, credentials, duties, analytics, training, governance, and change management.	https://www.usajobs.gov/job/878124700
12	Exclude	X	Live government page	Closing date 2026-08-10; opening date requires final capture	PharmD/BS Pharmacy and pharmacist license	Uses population health tools for risk mitigation	Audit disproved the interim label: this is a pain/opioid-safety program-manager position, not a pharmacy-informatics or AI job.	https://www.usajobs.gov/job/878370500
13	Include	B1	Employer Workday record indexed	Exact: 2026-06-19	Graduate of an ACPE-accredited School of Pharmacy with a Bachelor of Science in Pharmacy degree or higher	Business intelligence, analytics, and pharmacy informatics	Date, pharmacy education, title and technology focus corroborated through the employer record.	https://uasys.wd5.myworkdayjobs.com/en-US/UASYS/job/Pharmacy-Informatics-Analyst_R0087138-1
14	Include; template sensitivity flag	B1	Employer Workday record indexed	Exact date NR; retrieved 2026-08-09	Clinical pharmacist requisition	EHRs, pharmacy informatics systems, medication automation and CDS optimization	Genuine employer record; standardized AdventHealth competency language counted once in template-collapsed analysis.	https://adventhealth.wd12.myworkdayjobs.com/en-US/AH_External_Career_Site/job/Clinical-Pharmacist-Nonexempt_R-0369605-1
15	Include; template sensitivity flag	B1	Employer Workday record indexed	Relative date displayed; exact date NR; retrieved 2026-08-09	Pharmacist requisition	EHR/pharmacy informatics, medication automation and clinical decision support	Genuine employer record; standardized competency language flagged for template collapse.	https://adventhealth.wd12.myworkdayjobs.com/en-US/AH_External_Career_Site/job/Pharmacist_R-0343323-1
16	Include	B1	Employer Workday record indexed	Exact: 2026-07-29	Inpatient Clinical Pharmacist requisition	EHRs, pharmacy informatics systems, and medication automation	Exact date, role identity and technology competency are corroborated; shared-template sensitivity flag applies.	https://adventhealth.wd12.myworkdayjobs.com/ah_external_career_site/job/adventhealth-la-grange-mem/inpatient-clinical-pharmacist_r-0385587
17	Include; template sensitivity flag	B1	Employer Workday record indexed	Exact date NR; retrieved 2026-08-09	Clinical Pharmacist Part-Time requisition	EHRs, pharmacy informatics systems, medication automation and CDS	Genuine employer record; standardized competency language flagged for template collapse.	https://adventhealth.wd12.myworkdayjobs.com/en-US/AH_External_Career_Site/job/Clinical-Pharmacist--Part-Time_R-0377061
18	Include; template sensitivity flag	B2	ACCP professional job-board record	Exact date NR; retrieved 2026-08-09	Advanced specialty clinical pharmacist role	EHR/informatics, automation, CDS, medication safety and teaching	Professional-board evidence is adequate for inclusion but employer/date corroboration is limited; analyze separately from B1.	https://careers.accp.com/job/advanced-heart-failure-cardiac-transplant-clinical-pharmacist-orlando-florida-70050
19	Include	B1	Employer Workday record and independent full-text mirror corroborated	Reposted in 2026; exact original date pending	Graduate of an accredited College of Pharmacy; Kentucky/Indiana license or eligibility	IT, automation systems, medication-use process, implementation, integration, training and system changes	Credentials and detailed duties corroborated; exact original posting date remains NR rather than inferred.	https://bhs.wd1.myworkdayjobs.com/en-US/careers/job/System-Informatics-Pharmacist-Specialist---Remote-from-IN-or-KY_R26099483
20	Include	B1	Employer Workday record indexed	Exact: 2026-07-29	Clinical Informatics Pharmacist requisition	Primary subject-matter expert for pharmacy clinical information systems	Employer, requisition, exact date, pharmacist identity and informatics function corroborated.	https://umiami.wd1.myworkdayjobs.com/en-US/UMCareerStaff/job/Clinical-Pharmacist---IT-Systems---Full-Time_R100098946
21	Include	B1	Live employer career page	Exact date not displayed in captured text	Informatics Pharmacist role; licensure details require expanded capture	IT components connected to ordering, dispensing, or administration of medications	Employer page confirms requisition R-10977, duties, location, and $158,288-$237,432 salary.	https://www.overlakecareers.org/job/informatics-pharmacist-clinical-specialist-pharmacy-us-wa-bellevue-1035-116th-ave-ne-buildin-r-10977-49/
22	Include	B1	Accessible employer-hosted career page	Current in August 2026; exact date not displayed	Pharmacist; Louisiana Board of Pharmacy licensure corroborated	Build, implementation, integration and maintenance of automation and technology	Employer page confirms the role and duties; date is recorded as NR/retrieved rather than inferred.	https://lcmchealth.wd1.myworkdayjobs.com/LCMCHealth/job/LCMC-Health-Corporate/XMLNAME-340B-Clinical-Informatics-Pharmacist_JR0039574-1

ID	Audit decision	Evidence grade	Page status 2026-08-09	Posting date status	Credential evidence (short source excerpt)	Technology evidence (short source excerpt)	Audit finding	Clickable source
23	Include	B1	Employer Workday record indexed	Current August 2026; exact date not captured	Current Indiana Pharmacist License or eligibility	Clinical pharmacy informatics coordination	Employer, requisition, licensure and informatics responsibility corroborated; exact date remains NR.	https://bhs.wd1.myworkdayjobs.com/careers/job/New-Albany-IN/Pharmacy-Informatics-Coordinator_R26104886
24	Include	B1	Employer Workday record plus independent indexed copy	Exact date NR; retrieved 2026-08-09	Clinical Informatics Pharmacist requisition	Clinical leadership for complex implementation; pharmacy informatics, automation and controlled-substance support	Employer requisition R00047937 and job function corroborated; exact posting date remains NR.	https://ummc.wd5.myworkdayjobs.com/en-US/umccareers/job/jackson-ms---main-campus/clinical-informatics-pharmacist---automation-and-narcotic-support---full-time_r00047937-2
25	Include	B1	Employer Oracle record plus independent full-text indexes	Exact: 2026-01-13	Clinical Informatics Pharmacist Specialist	Clinical drug and medical-device data translated into decision-support knowledge	Employer, requisition, title, date and informatics function corroborated.	https://eevd.fa.us6.oraclecloud.com/hcmUI/CandidateExperience/en/sites/FDB/job/2025396
26	Include	B1	Closed employer record/archived text corroborated	Closed: 2026-05-14	Clinical Informatics Pharmacist role	EHR medication content, order sets, CDS, workflow, testing, validation and implementation	Detailed employer-originated duties, compensation and closing date are available; opening date remains NR.	https://imh.wd108.myworkdayjobs.com/en-US/IntermountainCareers/job/Clinical-Informatics-Pharmacist_R170123
27	Include	B1	Employer page indexed	Exact: 2026-05-18	PharmD/equivalent and active pharmacist license	Epic configuration, smart infusion-pump integration, system build and informatics leadership	Employer, date, credentials and core duties corroborated.	https://careers.mountsinai.org/jobs/3038168?lang=en-us
28	Include	A1	Live recruiter page	Not reported	Active pharmacist license in good standing	Analyze and review AI-generated content related to prescriptions, drug interactions, and patient education	Primary recruiter page directly confirms pharmacist licensure and detailed AI-training, safety, ethics, privacy, and communication duties.	https://www.ryzlabs.com/jobs/pharmacist-ai-trainer-remote-41d28
29	Include; geographic-mirror cluster collapsed	A2	LinkedIn full text plus concordant indexed copies	Relative date displayed; retrieved 2026-08-10	Existing clinical pharmacist; Medication Management (PharmD) expertise	Provide complex healthcare problems; evaluate AI logic, accuracy, and performance; ensure medical accuracy and response quality	One nationwide DataAnnotation opportunity; state mirrors collapsed; U.S.-only contractor.	https://www.linkedin.com/jobs/view/clinical-pharmacist-ai-trainer-at-dataannotation-4377553542
30	Include; geographic-mirror cluster collapsed	A1	Live original Greenhouse employer page plus indexed copies	Exact date available on one mirror: 2026-04-01; retrieved 2026-08-10	Licensed pharmacist/PharmD or RPh in good standing; recent practice experience	Evaluate pharmaceutical scenarios for accuracy, safety and reasoning; compare outputs; justify preferred answer; write exemplars and structured feedback	One Prolific expert-pool opportunity; numerous U.S. city mirrors collapsed.	https://job-boards.eu.greenhouse.io/prolificacademicltd/jobs/4869747101
31	Include	A2	LinkedIn full text; closed posting	Relative: 8 months; retrieved 2026-08-10	Clinical pharmacist; pharmacotherapy, medication-management and clinical-decision expertise	Train, validate and review AI; analyze cases/labs; evaluate outputs; give iterative feedback; create datasets, guidelines and protocols	Remote contract; North American version retained; duplicate micro1 title variants treated as one template cluster.	https://www.linkedin.com/jobs/view/clinical-pharmacist-ai-trainer-at-micro1-4340109569
32	Include with scope flag	A2	LinkedIn full text; closed posting	Relative: 4–5 months; retrieved 2026-08-10	Advanced healthcare credential including PharmD; ≥5 years healthcare experience	Write/refine prompts; evaluate accuracy, reasoning and completeness; verify claims; score models using frameworks/taxonomies	U.S. candidates accepted, but role is healthcare-wide rather than pharmacist-exclusive; sensitivity flag retained.	https://www.linkedin.com/jobs/view/healthcare-expert-remote-at-taskify-ai-4379701904
33	Include with scope flag	A1	Live original Novartis employer page	Exact: 2026-06-26	PhD or PharmD with pharmacokinetics/drug-metabolism background	Lead AI-enabled clinical-pharmacology strategy; develop AI use cases for PK/PD, dosing, regulatory deliverables and automation	Conventional pharmaceutical-industry role; pharmacist-eligible rather than pharmacist-exclusive.	https://www.novartis.com/careers/career-search/job/details/req-10081408-principal-scientist-iisenior-principal-scientist-pk-sciences-clinical-pharmacology-artificial-intelligence-ai-lead-dual-level-posting
34	Exclude—non-U.S. geography	X	LinkedIn full text	Retrieved 2026-08-10	India-specific pharmacy qualifications and registration	AI training, drug-safety evaluation, RLHF, annotation and red-team testing	Page location metadata conflicted with India/APAC qualifications and duties; excluded from U.S. sample.	https://www.linkedin.com/jobs/view/pharmacist-%E2%80%94-medical-ai-trainer-drug-safety-evaluator-at-sourcebae-4390823569

Supplementary Table S4. AI Workforce-Readiness Competency Framework

AI workforce-readiness competency	Definition	Observed workforce evidence	Required pharmacist foundation	Illustrative educational performance
AI-output evaluation and validation	Systematically determine whether an AI-generated pharmacy output is accurate, complete, clinically reasoned, appropriately uncertain, and safe; accept, correct, escalate, or reject it.	Output review; medication-safety validation; corrective reasoning; comparative adjudication; evidence and quantitative verification.	Pharmacotherapy, medication safety, calculations, PK/PD, evidence appraisal, and patient-specific clinical judgment.	Audit an AI-generated therapeutic recommendation, document consequential errors or omissions, and provide a defensible corrected response.
AI systems and workflow literacy	Explain and trace how inputs, prompts, data or retrieval sources, model behavior, tools, and human oversight combine to produce an AI output, including where failure may occur.	Recognition of hallucination, inconsistency, missing context, bias, data limitations, and the distinction between model output and verified clinical reasoning.	Medication-use processes, health informatics, clinical decision support, data provenance, and professional accountability.	Map an AI-supported medication workflow, identify failure points and missing controls, and explain why a plausible output may still be unsafe.
Prompt and evaluation-task design	Construct and refine prompts, cases, test scenarios, expected-answer criteria, and reference materials that elicit useful outputs and expose clinically consequential failure modes.	Prompt construction; clinical case and problem design; adversarial testing; dataset, protocol, exemplar, and gold-standard development.	Clinical problem formulation, patient-context specification, case construction, and articulation of acceptable therapeutic reasoning.	Design a prompt and case set in which clinically consequential variables are varied, with expected responses and explicit failure criteria.
AI performance assessment and improvement	Apply reproducible rubrics, benchmarks, comparative review, calibration, and repeated testing to characterize performance patterns and provide structured feedback for improvement.	Scoring frameworks and taxonomies; paired-output comparisons; error-pattern analysis; model-progress measurement; iterative feedback and reference-set curation.	Quality improvement, assessment design, evidence standards, severity-of-harm judgment, and interrater reasoning.	Use a defined rubric across multiple cases or model versions, analyze recurring errors and variability, and recommend evidence-grounded improvements.
Responsible AI implementation and governance	Determine whether AI is appropriate for a pharmacy problem; integrate it into a workflow with privacy, fairness, safety, monitoring, human-review, escalation, and accountability controls.	AI use-case identification; clinical and educational workflow integration; ethics, privacy, bias, governance, regulation, monitoring, and clinical-to-technical translation.	Medication-use systems, implementation science, ethics, regulation, patient safety, communication, and organizational leadership.	Conduct a preimplementation assessment defining intended use, evidence threshold, human authority, escalation rules, monitoring indicators, and stop criteria.
